# Severe acute respiratory coronavirus virus 2 (SARS-CoV-2) infection in asymptomatic vaccinated healthcare workers

**DOI:** 10.1017/ice.2021.224

**Published:** 2021-05-10

**Authors:** Verena Damiani, Domitilla Mandatori, Simone De Fabritiis, Sandra Bibbò, Rossella Ferrante, Fabrizio Di Giuseppe, Anna Giulia Ruggieri, Carola Di Camillo, Carlotta Buccolini, Dora Pizzi, Paolo Fazii, Liborio Stuppia, Vincenzo De Laurenzi

**Affiliations:** 1 Dipartimento di Tecnologie Innovative in Medicina & Odontoiatria, G. d’Annunzio University of Chieti-Pescara, Italy; 2 Center for Advanced Studies and Technology (CAST), G. d’Annunzio University of Chieti-Pescara, Italy; 3 Dipartimento di Scienze Mediche, Orali e Biotecnologiche, G. d’Annunzio University of Chieti-Pescara, Italy; 4 Dipartimento di Scienze Psicologiche, della Salute e del Territorio, G. d’Annunzio University of Chieti-Pescara, Italy; 5 Dipartimento di Microbiologia e Virologia, University of Chieti-Pescara, S. Spirito Hospital of Pescara, Pescara, Italy

Severe acute respiratory syndrome coronavirus 2 (SARS-CoV-2) infection has caused >2.7 million deaths worldwide,^1^ devasting health systems end economies worldwide. Since the first months of the pandemic, a rapid and massive effort has been performed by the scientific community to develop a safe and effective vaccine against SARS-CoV-2.^2^ In Italy, the first population to receive the vaccine was healthcare workers (HCWs) who are principally exposed to SARS-CoV-2 infection during the management of coronavirus disease 2019 (COVID-19) patients.^3^ Several doubts remain concerning the neutralizing properties of antibodies produced after vaccination.^4^ Moreover, little is known about transient infections in vaccinated individuals who therefore could be potential carriers of the disease.^5^ Finally, it is important to understand the actual efficacy of the approved vaccines against SARS-CoV-2 variants, which have led to enhanced virus transmissibility, morbidity, and mortality.^6,7^

Here, we report several asymptomatic and vaccinated HCWs who tested positive for SARS-CoV-2 during surveillance testing.

## Methods

### Samples

Approximately 500 nasopharyngeal swab specimens of HCWs and hospitalized patients were collected at the Hospital Ss. Annunziata of Chieti, Italy, and analyzed by the Laboratory of Molecular Genetics Test Diagnosis COVID-19 of the Center for Advanced Studies and Technology (CAST) at Gabriele d’Annunzio University of Chieti-Pescara, Italy.

### RNA extraction and qRT-PCR

RNA was extracted from nasopharyngeal specimens, using the MagMAX Viral/Pathogen II Nucleic Acid Isolation Kit on the automated KingFisher processor (Thermo Fisher Scientific, Waltham, MA). The extracted RNA underwent real-time reverse transcription polymerase chain reaction (qRT-PCR) with 2 commercial kits: the TaqPath COVID-19 CE-IVD RT-PCR Kit assay (Thermo Fisher Scientific) and Allplex SARS-CoV-2 Variants I Assay (Seegene, Korea).

### Next-generation sequencing (NGS)

For whole viral genome sequencing, total RNA was reverse transcribed using Invitrogen SuperScript VILO cDNA Synthesis Kit (Thermo Fisher Scientific). cDNA libraries were prepared using the Ion AmpliSeq SARS-CoV-2 Research Panel (Thermo Fisher Scientific). Sequencing was performed on the Ion GeneStudio S5 System (Thermo Fisher Scientific). The consensus sequences were aligned with the Wuhan-Hu reference SARS-CoV-2 genome using the Torrent Suite platform (Thermo Fisher Scientific). For phylogenetic analysis the whole-genome sequences of the isolates were uploaded on Pangolin COVID-19 Lineage Assigner.^8^

## Results

From January to March 2021, we were informed that among those who tested positive for SARS-CoV-2, 7 were HCWs who had received the BNT162b2 vaccination (Table 1). All were contacted and gave informed consent for this study.

**Table 1.** Vaccination Status, qRT-PCR Results, and NGS Summary

ID	Date of Vaccination		Date of Testing		Results of TaqPath SARS-CoV-2 Positive qRT-PCR Test, Ct Value			Lineage	Detection Method	Mutations		
	First Dose	Second Dose	Positive	Negative	ORF1 ab	N	S			E484K	N501Y	Δ69/70
HCW 1	13/01/21	…	19/01/21	20/01/21	28.949	29.201	ND	B.1.1.7	NGS	No	Yes	Yes
HCW 2	05/01/21	28/01/21	01/02/21	02/02/21	31.656	31.720	ND	B.1.1.7	NGS	No	Yes	Yes
HCW 3	04/01/21	26/01/21	01/03/21	12/03/21	18.318	17.463	ND	B.1.1.7	NGS	No	Yes	Yes
HCW 4	10/01/21	31/01/21	06/02/21	17/02/21	35.823	34.976	ND	B.1.1.7	Allplex^a^	No	Yes	Yes
HCW 5	13/01/21	03/02/21	06/02/21	17/02/21	36.236	36.911	ND	B.1.1.7	Allplex	No	Yes	Yes
HCW 6	03/01/21	24/01/21	16/02/21	17/02/21	31.645	31.213	ND	B.1.1.7	Allplex	No	Yes	Yes
HCW 7	05/01/21	26/01/21	02/03/21	13/03/21	15.894	15.866	ND	B.1.1.7	Allplex	No	Yes	Yes

Note. qRT-PCR, real-time reverse transcription polymerase chain reaction; NGS, next-generation sequencing of the entire viral genome; ID, study identification number; HCW, healthcare worker; Ct, cycle threshold; ORF, open reading frame; N, nucleocapsid; S, spike; ND, Ct value not determined.

a Allplex indicates that the presence of Δ69/70, N501Y and E484K were evaluated using Allplex SARS-CoV-2 variants I assay.

Of these 7 HCWs, 6 had received both doses of the vaccine and 1 had received only the first dose. HCWs 2, 4, and 5 received positive SARS-COV-2 results between 3 and 8 days after receiving the second dose. The remaining 3 cases (HCWs 3, 6, and 7) received positive results between 23 and 36 days after the administration of the second dose of vaccine.

All HCWs were completely asymptomatic and had undergone testing in a routine surveillance schedule for HCWs. In 3 cases, we were able to retest the HCWs the day after the positive qRT-PCR result and show that they were already negative. The remaining cases were tested after 10 days, and all were negative for SARS-CoV-2 infection.

In all 7 cases, molecular tests showed the presence of the Δ69/70 deletion of the S gene found in the B.1.1.7 lineage known as the UK variant, indicated by the TaqPath kit, which fails to amplify the S gene in the presence of the Δ69/70 deletion.^9^

The NGSs of the viral genomes of HCWs 1, 2, and 3 confirmed that the infections belonged to the B.1.1.7 lineage. The remaining cases (HCWs 4, 5, 6, and 7) were tested using the Allplex Assay, which confirmed the presence of the Δ69/70 deletion and of the N501Y mutation, thus confirming that these cases belonged to the B.1.1.7 lineage.

## Discussion

Data collected in this study confirm that infection is possible following vaccination. In 1 case, this was expected because the infection occurred only a few days after the administration of the first dose of vaccine, likely before the production of antibodies. Indeed, it has been reported that neutralizing antibody levels start to increase 8 days after the first dose of vaccine reaching the peak 2 weeks after the boost of the second dose.^4^ The infection that occurred in 3 HCWs a few days after the administration of the second dose may have been due to the weak titer of neutralizing antibodies produced.

The last 3 cases show that some individuals can still be infected after having completed the vaccination schedule and after sufficient time has passed for the peak of antibody production to occur. Unfortunately, we were not able to evaluate antibody levels at the time of infection; therefore, we were unable to correlate infection with low antibody titer.

Finally, all of the cases we observed in this report were due B.1.1.7 lineage infection. Although we cannot draw conclusions due to the limited numbers of cases observed so far, we can confirm that the vaccine has an effect on this variant because none of the infected individuals showed symptoms and all rapidly became SARS-CoV-2 negative.

In conclusion, although further data and observations of larger cohorts are needed, we strongly believe that continued attention should be devoted to the problem of infection in vaccinated people. Our data demonstrate that some people can have transient asymptomatic infections following vaccination and can therefore be potentially infectious, thus suggesting the necessity of precaution to be maintained particularly for HCWs to avoid spreading the virus, particularly among hospitalized people.
